# Dr. Kennedy on Life and Mind
*Extracted from an "Introductory Lecture delivered at a meeting of the members of the *Warwick* and *Leamington* Literary and Scientific Institution, holden at the Lecture-room, Warwick, June 8th, 1829, by James M. S. Kennedy, M.D., of Ashby-de-la-Zouch."


**Published:** 1830-07-01

**Authors:** 


### Periscope;

OR,

### CIRCUMSPECTIVE REVIEW.

"Ore trahit quodcunque potest, atque addit acervo."

I.

### Dr. Kennedy on Life and Mind.*

Zoonomy may be accounted the science of *living* things—vegetables, animals, man, and of their distinctive attributes —organization, vitality, and mind.

Organization implies life; and, in animals, is associated with mind. Physiologists give the term a two-fold signification. Under the first, it expresses the *act* of eliciting appropriate particles from the substances of nutrition, and applying them to their destined ends—the formation, sustenance, renewal, and propagation of living structure; under the second, it denotes the *state* of such particles, so formed and applied as to constitute an organ or living instrument, by the composition of its elementary principles. Hence, as an *effective process*, it imports the separation of organizable atoms or essences from the blood by means of a secreting function, and of ultimately adapting them to their determinate uses through the instrumentality of vital absorption: and, as a *constituent state*, it has reference to the circumstances of animal texture thus constructed. Organization, therefore, implies the aggregate of those qualities which distinguish the living from inanimate formations.

Vitality is the *action* of life co-efficient with organic instruments. Life forms a constituent element in every organized thing that executes motion: it is itself a substantial entity, an operative principle exercising positive agency, causing manifest effects from which its substantiality is deducible: it is, indeed, the source of all organic *action* and was communicated, in the beginning, by the creative inspiration of the Almighty: its operative manifestations are perceptible; but, as with the elemental principles of light and caloric, philosophers are utterly ignorant of its nature and essence: the divine oracles have not revealed these, and hitherto they have eluded observation as well as scientific research. That incomprehensible principle, then, which was thus imparted to the first of all animate beings, and to the first of the human kind, and made communicable through the processes of reproduction, to the latest born of every race; that principle which gives to vegetables the power of converting the elements of inert matter into organized structures; that principle which, in animals, by the unceasing agency of its own peculiar vehicle —*arterial blood* — transmits to every organic texture the germs of its essential and vital attributes, *is* LIFE: the equal tenour of the operations of life, maintains health; their derangement originates disease, by the fatal ascendency of which, *whatever lives* is doomed to languish, to sicken, and to die.

Arterial blood is an instrument or vehicle only: it transmits or imparts, but *is not*, the principle of life: it is the diffusive source of organization, vitality, and mind: it pervades and in-

* Extracted from an "Introductory Lecture delivered at a meeting of the members of the *Warwick* and *Leamington* Literary and Scientific Institution, holden at the Lecture-room, Warwick, June 8th, 1829, by James M. S. Kennedy, M.D., of Ashby-de-la-Zouch."

vigorates every portion of the living machine; furnishes to each *new* being all the material and mental elements which the animal organization originally comprehended, and by which it is perpetually sustained. From this blood the *nervous*, as well as all other structures, is primarily elaborated; and this structure, in being made the organic depository and instrument of mind, has been qualified to discharge the exquisite office of manifesting the innumerable modifications of feeling, sentiment, and intelligence. Hence it is, that arterial blood, as a communicative instrument, gives, and upholds, and repairs the essential and vital elements of animals; and nervous structure, as an instrument also, supplies unceasingly their active and sentient energies.

Philosophy and revelation represent man as a superlative being, in whom the qualities of a mortal nature are associated with the attributes of immortality. This pre-eminence of constitution is innate, and forms his distinguishing character: it results from his possession of organization and life connected, by inexplicable ties, with a transcendancy of mental endowment; and, as it contributes essentially to the vigour and dignity of his progressive states, it exercises important influences on the circumstances of his ultimate destiny. In accordance, therefore, with the benevolence of divine wisdom and power, the Creator has furnished him with organs, adapted in all respects to the complete discharge of those vital and mental functions, on which the integrity and transmission of his exquisite economy depend. Nevertheless, in being exposed to sustain impressions from the manifold and ever-varying agents which tend incessantly to change the modifications of existence throughout the universe, the human fabric carries, in each of its systems, the elements of health in conjunction with a liability to disease.

Man was *one*, at the beginning; and, from the primogenial sire, have sprung every individual, tribe, and nation, in every region of the habitable world. He was created perfect in all his endowments; and, by this provision, was made capable of performing, intuitively and without experience, the admirable functions of life, sense and intelligence. Organization, therefore, and life and mind arose originally a matured constructure, elaborated by the Creator's plastic hand; and, thenceforward, have been maintained and transmitted by the vital actions of organs.

Mind is propagated by the same genial act which renews the origins of organic structure and life: it implies the co-existence and co-operation of its own, with the organic and vital attributes, and thus constitutes the characteristic distinction whereby the animal surpasses in power and dignity, every other modification of nature. This doctrine of the elements of mind being transmitted by parents to their progeny, should evidently be regarded as a postulate merely; and, by consequence not capable of inductive demonstration: nevertheless, by receiving the assumption, we shall extricate ourselves from the inducements to adopt another postulate equally indemonstrable and beset moreover with manifest absurdity — namely, that of admitting a necessity for the continuous exercise of Almighty power in the creation of a new mind or soul for every new being, however impure its source.

The mind's *essence* is altogether indeterminable, but its individuality is certain and susceptible of philosophical investigation. It consists of an aggregate or system of faculties, every *one* of which exercises its functions through *one* of a corresponding aggregate or system of corporeal organs; and, by this arrangement, its economy is governed by the absolute physiological law—that one organ never performs more than one distinct function. Animals, therefore, of every kind and in all their manifold gradations, do possess mental endowment; but, in each, the degrees of this can be perceived and appreciated only by its manifestations and the exquisiteness of the corporeal instruments, by the agency of which these manifestations are exhibited.

Physicians have been accustomed, from observation of disorder in the mental *manifestations*, to regard the

*mind itself* as being susceptible of change and decay, of entering indeed into all the morbid conditions which the illimitable state—Insanity—comprehends: but, since we know not the mind's peculiar essence, the best philosophy would be—to consider such disorder as connected with different conditions of the material organs by means of which its operations are felt and made apparent: these organs certainly do admit of growth, maturity and decay; they sustain progressive changes, and have likewise their functions altered by the influences of disease: the imputation of disease to the *rational* mind, implies its liability to death and decomposition, and chills our whole nature by extinguishing the hope of immortality.

While, then, the mere vegetable exercises its intuitive ability to select the nutriment most proper for its own conditions; while the irrational animal feels and desires, enjoys a brief existence, and passes into oblivion: man not only has, in more perfect endowment, the faculties assigned to other living beings, but he stands exalted above all creatures in the possession of a moral nature, susceptible of direction by judging and reflecting powers. Faculties peculiar to himself, inspire him with a disposition to the practice of justice and charity: an innate sentiment of religion prompts him to worship a *Supreme Being*; and, guided by its higher energies, his intelligence discovers that He who made the earth and ocean, the starry firmament, and the everlasting sun, He is God. Its own consciousness of an inherent longing after immortality, carries his mind forward in endless progression, into periods of ever-during time: an instinctive tendency to leave this world with all its enjoyments, to spring forward into a far distant futurity, and to expatiate, even in imagination, amid the scenes of an eternity to come, gives to man the expectant assurance that he is formed for a more glorious destiny than to perish for ever in the grave.

